# An Account of a Successful Method of Reducing the Funis, in Cases in Which It Comes down before the Head of the Fœtus

**Published:** 1786

**Authors:** Richard Croft

**Affiliations:** Surgeon at Tutbury, in Staffordshire


VIII. *An Account of a succeſsful Method of reducing the Funis, in Caſes in which it comes down before the Head of the Fœtus. By Mr.* Richard Croft, *Surgeon at Tutbury, in Staffordſhire. Communicated in a Letter to Dr.* Denman, *and by him to Dr.* Simmons.

NOT long ago I attended in a caſe of midwifery where there was a prolapſus of the funis before the preſenting part, or head, of the child.

From the woman's information I found, that in former labours, ſhe had four times undergone the operation of turning, by an expert practitioner, who had been only able to ſave one child. On hearing this, it occurred to me, ſhe muſt have a narrow Pelvis, but finding that not to be the caſe, the membranes being now broken and the pulſation in the funis pretty ſtrong, my firſt buſineſs was to attempt the reduction of the funis.

As the head was then entering the brim of the pelvis, I very eaſily accompliſhed this operation, according to the directions of our books and teachers of Midwifery, by wrapping part of the funis in a rag. In ſimilar caſes I had frequently performed the ſame operation, in prefepreference to turning; but I am ſorry to ſay, not often with the ſucceſs I wiſhed. In this caſe, as I had found in many others, a pain or two brought the funis down again. After being repeatedly foiled, and finding the pulſation grow rather weak, I determined on the operation of turning. With a full intention of performing it, I introduced my hand into the Uterus; but, juſt as I got to the feet of the child, it ſtruck me that there was a probability, if not a certainty, of retaining the funis above the head by hanging it over one of the child's legs. This I accordingly did; the woman lay about an hour without much pain, in conſequence of an opiate I had given her, and eight or ten hours after the return of her pains before the birth, the funis never came down, and I had the ſatisfaction of delivering her of a living child.

The ſucceſs of this operation determined me to put it in practice in another caſe of the ſame kind to which I was called ſoon after. Here the head was farther advanced into the pelvis, but not far enough to admit the uſe of the forceps: a ſkilful midwife, who was attending, had reduced the funis in the uſual manner, and once after my arrival, when ſhe thought it would not again come down. But this failing, as well as her former attempts, I immediately ſet about my operation. In this caſe I found more difficulty than before in getting my hand beyond the head, but when I had ſucceeded in that, the operation terminated as favourably as in the former caſe.

